# Blindness Caused by the Use of Sulph. Quinine

**Published:** 1847-02

**Authors:** John McLean

**Affiliations:** Prof. of Materia Medina in the Rush Medical College


*Blindness Caused by the Use of Sulph. Quinine.* By John McLean, M. D., Prof. of Materia Medica in the Rush Medical College. —Quinine when freely administered produces a species of intoxication, tinnitus aurium, a sense of fulness in the head, cephalagia, and other affections; and sometimes, although not so frequently, blindness, more or less lasting.

M. Trousseau, relates the case of a tailor, who, for the relief of a periodical asthma, took 48 grs. of the sulph. quinine, at one dose. In four hours he experienced ringing in the ears, dulness of the senses and vertigo; and in seven hours, he was blind and deaf, his mind wandered and he was unable to walk. These effects, for which no active medicine was administered, gave way spontaneously, during the night. A young girl at the "Hospital Cochin," in consequence of having taken freely of the sulph. quinine, became affected with amaurosis, which continued at the end of three weeks, notwithstanding appropriate and energetic means were employed for the restoration of her sight.

Dr. Rognetta, who claims for Rasori, priority in the use of quinine in acute rheumatism, "thinks, with the Italian physicians 'that the limits of tolerance should not be exceeded, and that beyond this, a species of poisoning may be induced, known by deafness, blindness, hallucinations, hæmaturia, &.'"

Blindness, although not so common as the other effects, is not unfrequently produced, and may be prolonged for months or even years. It is not, however, generally known, that such may be the result of this medicine, when given in large quantities. The following are some cases occurring in this place and immediate vicinity, which show that when thus administered it may produce blindness more or less permanent.

*Case* 1st. Mr. P. of the town of Barry, Jackson co., was in the year 1840 attacked with a low grade of remittent fever, the nature of which was such, as to cause the attending physician to administer the sulph. quinine in large and frequent doses. Sixteen grains, (as judged by sight,) were ordered every hour, and continued until nearly one ounce was taken. Before the quinine was discontinued, he became perfectly blind, which, with a slow and gradual amendment, continued during the first year. Later than this, I have not been positively informed in regard to the case, but should judge, from what indirect information I have reeeived, that his sight is not yet perfectly restored.

*Case* 2d. Mrs. B., of the town of Concord in this county, was, a few years since, reduced so low by the endemic fever of the country, that her life was despaired of. As a last resort large quantities of quinine were given, and while taking it she became blind, which continued for several weeks. As she recovered her health the blindness gave way, and her sight was finally restored. Not being acquainted with the particulars of this case, I can give but these few general outlines.

*Case* 3d. P. M. Everett, of this place, was, in the autumn of 1843, attacked with remittent fever, and in a few days became so greatly reduced, as to leave but slight hopes of his recovery. Sulph. Quinine was therefore prescribed, in doses averaging three grains, every hour, and was continued for three days. In a short time, he became deaf, and soon after so blind that he could not see a burning candle, when placed immediately before his eyes. The blindness took place on the third day, after the commencement of the free administration of the sulph. quinine. Previous to this, and at this time, his mind, (with the exception of occasional slight wanderings) appeared to be perfectly clear. After some weeks, his sight became partially restored, but continues more or less imperfect, even at the present time.

During the greater part of the first year, he could look steadily at the sun, without seeing it, or even any painful sensation being produced. When he first began to see sufficiently to read, which was in the course of the first year, he could perceive but a small luminous spot upon the paper, about one inch in diameter, within which he could distinguish letters, but all without this was cloudless and confusion. During this time, the pupils were very much dilated, and he could see objects at a distance much better than those near by. His sight has continued to improve, ever since; and at the present time, although quite imperfect, is sufficiently good to enable him to read and write, although with some difficulty. The pupils are still considerably dilated, and it is with great difficulty, that he can discern objects by twilight. The direct rays of the sun upon the head, produce pain there, accompanied with a painful sensation deep in the orbit of the eye, and a disordered vision. At the present time, exercise easily produces fatigue, by which his sight is much impaired.

*Case* 4th. In the month of April, 1846, Dr. R. of this place took in doses of six grs. each, three drachms of quinine in 36 hours; at the expiration of which time, he became perfectly blind. His hearing was somewhat blunted, although it did not, in degree, equal the blindness. On the two succeeding days, his sight, although very imperfect was considerably restored. Had he lived, the probability is, that this imperfect sight would, as in the former cases, have continued a considerable length of time.

*Remarks.*—We think it clear that the blindness in the foregoing cases was the effect of the quinine; for we see it in each, coming on suddenly during its administration in large quantities, and at a time, when no other medicine was given that would be likely to produce such results. Here, cause and effect appear to be closely connected, and are so plain, as scarcely to admit of the possibility of a doubt. From the symptoms accompanying the foregoing cases, we judge that the proximate cause of the blindness, was mainly an affection of the retina or optic nerve, producing amaurosis.

I have recorded the foregoing facts, with the hope that they might be the means of causing some useful suggestions, in relation to the physiological effect and administration of this medicine.

In connection with the foregoing, we might mention the case of Mr. B. Porter, of this town, who has had for sixteen years and upwards, amaurosis of the left eye, which he supposed to have been produced by the application of a strong subacetate of copper ointment to that side of the face, for the purpose of curing *Herpes circinatus*. As the ringworm gave way, the blindness came on.

About one year since, he suffered with a periodical neuralgia, for which I ordered 32 grs. of quinine to be taken in divided doses of 4 grs. each, every two hours. Under its influence, the neuralgia disappeared; and on the following day, he could see objects quite distinctly with the amaurotic eye, much better than ever before, since it first became diseased, and he was much elated with the thought of soon regaining its sight. He, however, took no more quinine, and in a few days, the benefit produced to that eye was entirely lost.

*Jackson*, (*Mich.*) Sept. 22, 1846.

*Illinois and Indiana Med. and Surg. Jour.*

